# Master Regulator Activating Transcription Factor 3 (ATF3) in Metabolic Homeostasis and Cancer

**DOI:** 10.3389/fendo.2020.00556

**Published:** 2020-08-14

**Authors:** Hui-Chen Ku, Ching-Feng Cheng

**Affiliations:** ^1^Department of Pediatrics, Taipei Tzu Chi Hospital, Buddhist Tzu Chi Medical Foundation, Taipei, Taiwan; ^2^Institute of Biomedical Sciences, Academia Sinica, Taipei, Taiwan; ^3^Department of Pediatrics, Tzu Chi University, Hualien, Taiwan

**Keywords:** ATF3, glucose metabolism, adipocyte, immunity, cancer

## Abstract

Activating transcription factor 3 (ATF3) is a stress-induced transcription factor that plays vital roles in modulating metabolism, immunity, and oncogenesis. ATF3 acts as a hub of the cellular adaptive-response network. Multiple extracellular signals, such as endoplasmic reticulum (ER) stress, cytokines, chemokines, and LPS, are connected to ATF3 induction. The function of ATF3 as a regulator of metabolism and immunity has recently sparked intense attention. In this review, we describe how ATF3 can act as both a transcriptional activator and a repressor. We then focus on the role of ATF3 and ATF3-regulated signals in modulating metabolism, immunity, and oncogenesis. The roles of ATF3 in glucose metabolism and adipose tissue regulation are also explored. Next, we summarize how ATF3 regulates immunity and maintains normal host defense. In addition, we elaborate on the roles of ATF3 as a regulator of prostate, breast, colon, lung, and liver cancers. Further understanding of how ATF3 regulates signaling pathways involved in glucose metabolism, adipocyte metabolism, immuno-responsiveness, and oncogenesis in various cancers, including prostate, breast, colon, lung, and liver cancers, is then provided. Finally, we demonstrate that ATF3 acts as a master regulator of metabolic homeostasis and, therefore, may be an appealing target for the treatment of metabolic dyshomeostasis, immune disorders, and various cancers.

## Introduction

Activating transcription factor 3 (ATF3) is a member of the ATF/cAMP response element-binding (CREB) family, which binds to the cyclic AMP response element (CRE) in numerous promoters with the consensus sequence TGACGTCA (1, 2). Besides, other sequences of ATF3 binding site on the target promoters are reported (3–6), including the promoter sequence of IL-12p40 (TCGACGTCT), CCL4 (TGACATCA), cyclin D1 (CCND1) (TAACGTCA), and growth arrest and DNA damage 153 (GADD153) (TTGCATCA). ATF3 can also interact with proteins via its basic leucine zipper (bZIP) domain and modulate cellular functions independently of its transcriptional activity (7, 8). The ATF3 gene consists of four exons that encode a 181-amino acid protein with a molecular weight of 22 kDa (7). ATF3 has been demonstrated to be a transcriptional repressor by forming a homodimer. In addition, the transcription factor cooperates with other ATF/CERB family proteins or CCAAT/enhancer-binding protein (C/EBP) family proteins to form heterodimers producing inhibitory or stimulatory effects depending on the condition of the cell and promoter (9, 10). The leucine zipper region of ATF3 is responsible for homodimers or heterodimers formation with other proteins containing the bZIP domain, including the activating protein 1 (AP-1), C/EBP, and musculoaponeurotic fibrosarcoma families of proteins (11). Hai et al. demonstrated that sequence analysis of the 5′-flanking promoter region of ATF3 exhibits numerous transcription factor-binding sites, such as AP-1, ATF/CRE, nuclear factor kappa-light-chain-enhancer of activated B cells (NF-κB), thus implying that ATF3 may be induced by stress signals, including UV radiation, cAMP, calcium influx, and cytokines (12, 13). The Myc/Myc associated factor X and E2 factor sites are implicated in the cell cycle regulatory function of the ATF3 promoter, thus suggesting that ATF3 expression may also be modulated in a cell cycle-dependent mode (12). ATF3 has been exhibited to be modified by post-translational modifications such as phosphorylation, acetylation, and SUMOylation. Lee et al. had shown that AM251, a cannabinoid antagonist, can inhibit the viability of hepatoma HepG2 cells via increased phosphorylation of JNK (c-Jun N-terminal kinase) and ATF3 (14). Furthermore, ATF3 acetylation by E1A-binding protein p300 (p300) is crucial for ATF3 binding to Gadd45β/γ promoters and consequently promotes Gadd45β/γ transcription and glomerular mesangial cells apoptosis induced by sublytic complement C5b-9 (15). Moreover, ATF3 can be SUMOylated. De-SUMOylation decreases ATF3-modulated CCND1 activation in PC3 and DU145 cells (16). Five truncated ATF3 isoforms, including ATF3Δzip, ATF3Δzip2 (ATF3Δzip2a and ATF3Δzip2b), ATF3Δzip2c, ATF3Δzip3, and ATF3b have been identified in different cellular systems (17, 18). ATF3Δzip was identified in HeLa cells after serum stimulation (9). ATF3Δzip2a and 2b were induced after treatment with various stress-associated stimuli in primary human umbilical vein endothelial cells (19). ATF3b was identified in untreated glucagon expressing pancreatic α cells and is implicated in modulating cAMP signaling of proglucagon transcription (20). Moreover, ATF3Δzip2c and ATF3Δzip3 were identified in HepG2 human hepatoma cells following amino acid deprivation (21). Except for ATF3b, other isoforms with deficient leucine zipper domain cannot form dimers to bind to DNA (17, 19, 22). ATF3 is commonly expressed at low levels in normal and quiescent cells. A strong body of evidence shows that ATF3 is an adaptive-response gene and that its expression is increased by numerous signals, including those triggered by genotoxic agents, cytokines, cell death-inducing agents, and physiological stresses (5, 11, 19, 23). Indeed, overwhelming evidence indicates that ATF3 plays an important role in metabolic regulation, immune responses, and oncogenesis. ATF3 regulates the expression of target genes based on the cell type and/or the nature of the stimuli. Given this background, we will discuss both cancer and metabolic disease together as cancer is suggested to disturb metabolic homeostasis and some metabolic alterations facilitate cancer initiation, progression, and remission. The purpose of this review is to summarize recent studies regarding the regulatory role of ATF3 in glucose metabolism, adipose tissue, immunity, and oncogenesis.

## Role of ATF3 in Glucose Metabolism

ATF3 is involved in glucose metabolism in a variety of organs and tissues, including the pancreas, liver, adipose tissue, hypothalamus, and heart. Several studies investigating the physiological roles of ATF3 in the pancreas have been carried out. However, these studies present crucial variances (20, 24–27). Hartman et al. for example, found that ATF3 is induced by proinflammatory cytokines, glucose, and palmitate in ß cells (25). ATF3 induction is partially mediated by the NF-κB and JNK/stress-activated protein kinase (SAPK) signaling pathways, which are two stress-induced pathways involved in diabetes. To further clarify the functional importance of ATF3 expression in islets, the transgenic mice expressing ATF3 under the control of the fragment from kb −2.7 to −1.7 of the pancreatic and duodenal homeobox 1 (PDX-1) promoter were generated (25). The PDX-1 promoter fragment was shown to target transgenes selectively in the developing islets and in β cells after birth. Furthermore, PDX-ATF3 transgenic mice develop abnormal islets. Islets deficient in ATF3 are moderately protected from cytokine- or nitric oxide-induced apoptosis (25). Wang et al. identified a new alternatively spliced isoform of ATF3 called ATF3b, which binds to the ATF/CRE site on the promoter of proglucagon gene, thus increasing its transcription in pancreatic α cells (20). Transgenic mice overexpressing ATF3 in the liver and pancreas driven by the transthyretin (TTR) promoter show glucose dyshomeostasis and perinatal lethality (28). Overexpression of ATF3 in the liver decreases steady-state mRNA levels of gluconeogenic enzymes, including phosphoenolpyruvate carboxykinase (PEPCK) and fructose-1,6-bisphosphatase (FBP). In addition, abnormal endocrine pancreas and decreased numbers of hormone-producing cells are observed in the pancreas of ATF3 transgenic mice (28). TTR-ATF3 transgenic mice show signs of liver dysfunction, such as increased serum alanine aminotransferase, aspartate transaminase, alkaline phosphatase, bilirubin, and bile acids (29). Mechanistically, ATF3 binds to an ATF/CRE site on the PEPCK promoter, thereby repressing its expression. These findings suggest that hepatic ATF3 represses gluconeogenesis (29). ATF3 expression is upregulated by low glucose and downregulated by high glucose in bothαTC-1.6 and ßTC3 cells. ATF3 increases the transcription of glucagon but not that of insulin (30). Zmuda et al. (31) showed that ATF3 KO islets are protected from three stress models, including islet isolation, treatment of inflammatory cytokines, and hypoxia. ATF3 increases various downstream target genes, such as pro-apoptotic genes (e.g., Noxa and Bnip3) and immunomodulating genes (e.g., TNFα, IL-1β, IL-6, and Ccl2). Furthermore, the islets of ATF3 KO mice show improved glucose homeostasis compared with those of wild-type (WT) mice after islet transplantation. Indeed, ATF3 KO islet grafts show downregulated expression of Noxa, Bnip3, TNFα, IL-1β, IL-6, and Ccl2 as well as reduced caspase 3 activation and macrophage infiltration (31). Lee et al. (32) found that pancreas- and hypothalamus-specific ATF3 KO mice exhibit improved glucose tolerance; however, the plasma glucagon and insulin levels of these mice are unaffected. The mice displayed a leaner phenotype, likely as a result of decreased food intake and enhanced energy expenditure (32). Mechanistically, ATF3 interacts with forkhead box-containing protein O subfamily 1 on the agouti-related protein (Agrp) promoter and upregulates Agrp transcription. Therefore, hypothalamic ATF3 is involved in the regulation of glucose and energy metabolism in mice through Agrp modulation (32). To investigate the mechanism for STZ-regulated diabetic liver, Kim et al. showed that streptozotocin (STZ)-induced harm to β-cells dependents on the signal transducer and activator of transcription 1 (STAT1), a pro-apoptotic gene (33). STZ treatment increases the expression of interferon-γ (IFNγ) and STAT1 in diabetic liver injury, which is accompanied by upregulated hepatic ATF3 expression. ATF3 stabilizes STAT1 by preventing the ubiquitination and proteasomal degradation of STAT1 in hepatocytes, thus suggesting that STZ induces IFN-γ, which is associated with the induction of ATF3, and IFN-γ-induced ATF3 may be involved in STAT1-regulated liver injury through STAT1 activation and its stabilization (33). Favre et al. demonstrated that downregulated expression of inducible cAMP early repressor in the white adipose tissue (WAT) of obese animals could cause constantly increase CREB activity, thus bringing about increased ATF3 expression and decreased Glut4 and adiponectin expression levels (34). However, studies by Zmuda et al. demonstrate that ATF3 plays a beneficial role in the high-fat diet (HFD)-induced diabetes and pancreatic ß-cell dysfunction. The authors found that WT mice fed an HFD for 12 weeks have better glucose tolerance but unchanged insulin resistance compared with global ATF3 KO mice (26); however, no differences in ß-cell mass and islet area or number were detected. Further analysis indicated that ATF3 binds to the insulin promoter in INS-1 ß-cells and primary islets to upregulate its expression (26). A recent study by Kalfon et al. indicated that HFD-fed cardiac-specific ATF3 knockout mice (ATF3-cKO) exhibit enhanced hyperglycemia and glucose intolerance (35).

**Figure 1:**
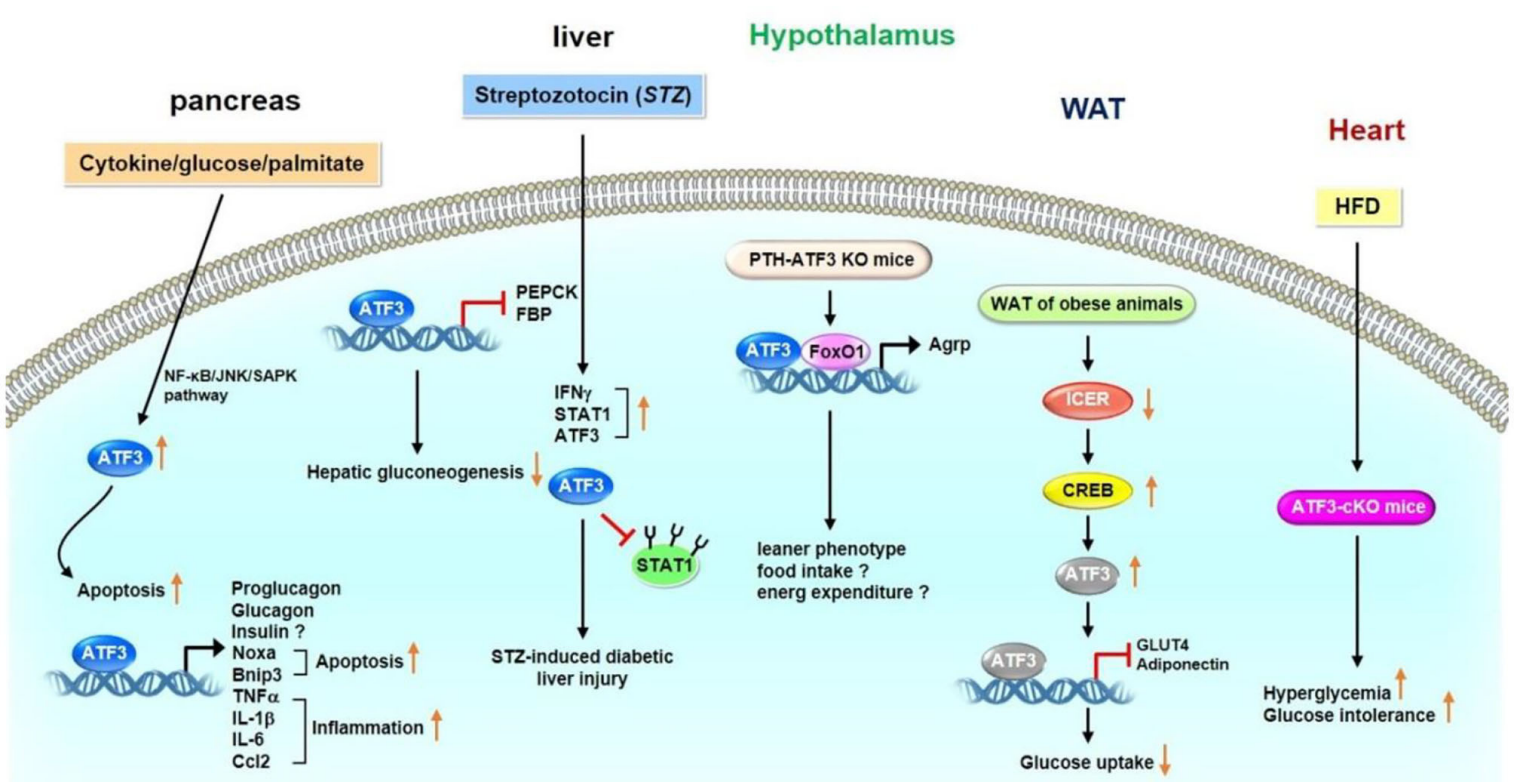
Role of ATF3 in glucose metabolism. ATF3 demonstrated to have different functions in various organs and tissues. In pancreas, ATF3 upregulates the expression levels of peptide hormone genes (proglucagon and glucagon), apoptosis-related genes (Noxa and Bnip3), and inflammation-related genes (TNFα, IL-1β, IL-6, and Ccl2). In liver, ATF3 decreases the expression levels of gluconeogenic enzymes (PEPCK and FBP) to inhibit gluconeogenesis. In addition, STZ treatment upregulates the expression levels of IFNγ, STAT1 and ATF3 in diabetic liver injury. ATF3 stabilized STAT1 by blocking ubiquitination and proteasomal degradation of STAT1 in hepatocytes. Loss of hypothalamic ATF3 decreases Agrp expression. These mice displayed a leaner phenotype possible as a result of decreased food intake and enhanced energy expenditure. ATF3 interacts with FoxO1 on Agrp promoter to upregulate its transcription. In adipose tissue, ATF3 may result in insulin resistance by downregulating Glut4 and adiponectin expression. In heart, HFD-fed ATF3-cKO mice show increased hyperglycemia and glucose intolerance. These observations indicate that ATF3 plays an important role in regulating glucose metabolism in various organs and tissues.

In summary, ATF3 has different functions in regulating glucose homeostasis. In the pancreas, ATF3 upregulates the expression levels of peptide hormone genes (e.g., proglucagon and glucagon), apoptosis-related genes (e.g., Noxa and Bnip3), and inflammation-related genes (e.g., TNFα, IL-1β, IL-6, and Ccl2). In the liver, ATF3 decreases the expression levels of gluconeogenic enzymes (e.g., PEPCK and FBP) to inhibit gluconeogenesis. ATF3 can also promote STZ-induced diabetic injury by stabilizing STAT1 expression in hepatic cells, and cardiac-specific HFD-fed ATF3-cKO mice show increased hyperglycemia and glucose intolerance. In adipose tissue, ATF3 may result in insulin resistance by downregulating Glut4 and adiponectin expression. ATF3 KO mice show reduced serum insulin levels with ß-cell dysfunction. In addition, loss of hypothalamic ATF3 decreases Agrp expression, and the ATF3 KO mice show a leaner phenotype with decreased food intake and enhanced energy expenditure. Taken together, these observations show that ATF3 may play an important, but a rather complex, role in regulating glucose metabolism through various organs and tissues (Figure 1). Further studies to elucidate the signaling pathways through which ATF3 inducers or inhibitors regulate the glucose metabolism in various organs and tissues are required.

## Role of ATF3 in Adipose Tissue

**Figure 2:**
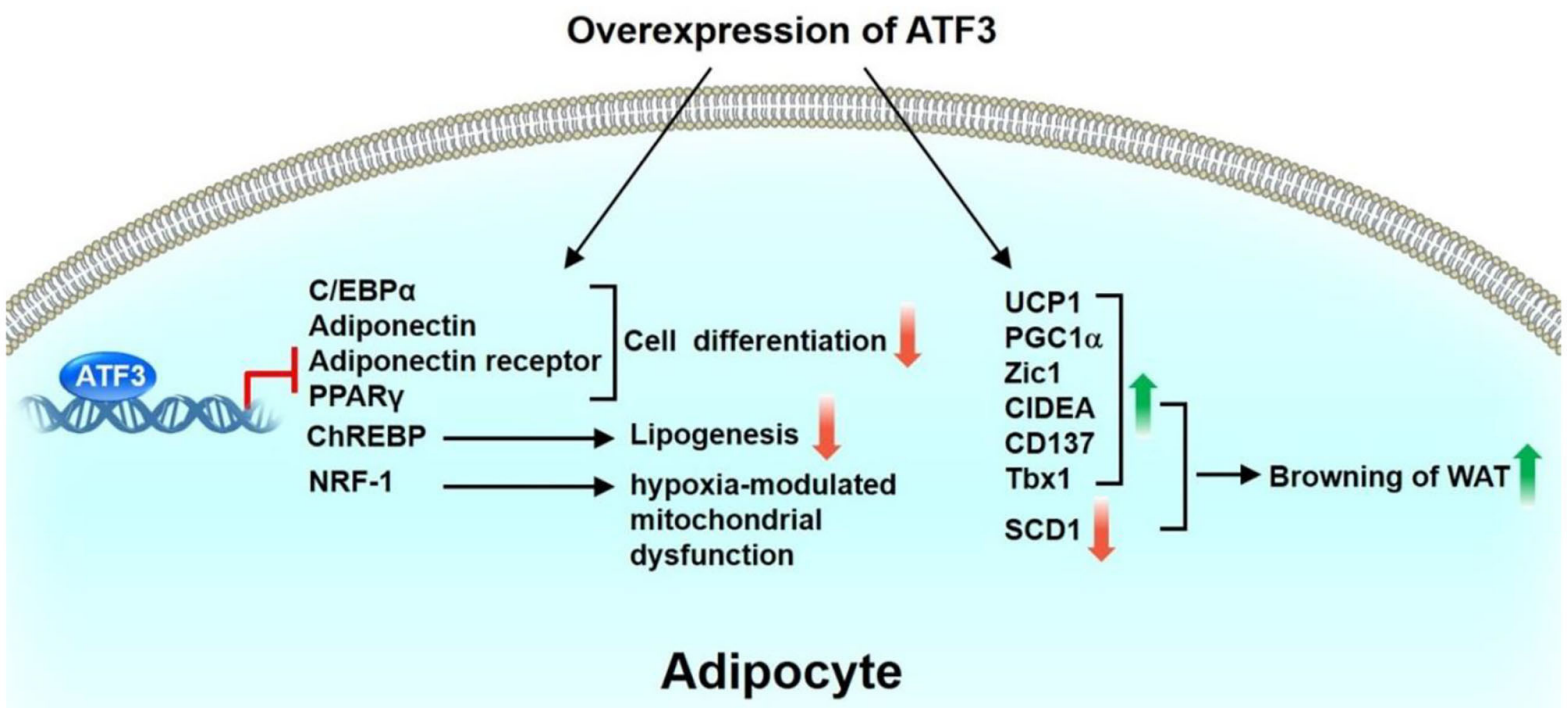
Role of ATF3 in adipose tissue. ATF3 inhibits adipocyte differentiation by negatively regulating the expression of adipogenic genes, including C/EBPα, adiponectin, adiponectin receptor, PPARγ. In addition, ATF3 suppresses ChREBP expression to inhibit lipogenesis of 3T3-L1 adipocytes. ATF3 is also involved in adipocyte hypoxia-modulated mitochondrial dysfunction in obesity via repression of NRF-1 expression. Overexpression of ATF3 induced transdifferentiation of white adipocytes to beige/brown adipocytes *in vitro* by upregulation of BAT/beige genes (UCP1, PGC1α, Zic1, CIDEA, CD137, Tbx1) and downregulation of SCD1. Taken together, these findings indicate that ATF3 plays a beneficial role in regulating adipogenesis, lipogenesis, and browning in adipocytes.

ATF3 is upregulated in the WAT of obese mice (36). ATF3 can downregulate the expression of C/EBPα (37) or PPARγ (38), resulting in suppression of adipocyte differentiation. Kim et al. found that ATF3 negatively regulates adiponectin gene expression (39). In addition, ATF3 suppresses the expression of adiponectin receptors in 3T3-L1 adipocyte cells and hepatocytes (40, 41). Mitochondrion-related genes, such as nuclear respiratory factor 1, peroxisome proliferator-activated receptor gamma coactivator 1-alpha, cytochrome C oxidase1, cytochrome C oxidase 2, and superoxide dismutase, are downregulated in the WAT of adipocyte-specific ATF3 transgenic mice compared with WT mice, thus suggesting the ATF3 is involved in adipocyte hypoxia-modulated mitochondrial dysfunction in obesity (36). Gold et al. showed that the deletion of ATF3 (ATF3^−/−^Apoe^−/−^) results in increased lipid body accumulation in macrophages compared with Apoe^−/−^ mice. Furthermore, ATF3 downregulates the transcription of the gene encoding cholesterol 25-hydroxylase (Ch25h), which enhances macrophage foam cell formation (42). Our recent study showed that ATF3 KO mice exhibit metabolic dyshomeostasis, including obesity and insulin resistance, compared with WT mice after HFD feeding (43). Overexpression of ATF3 induced the trans-differentiation of white adipocytes into beige/brown adipocytes *in vitro*. We further found that ST32da, a synthetic ATF3 inducer selected from an ATF3-specific promoter screening platform, enhances ATF3 expression to inhibit adipogenesis/lipogenesis and promote adipocyte browning by inhibiting the carbohydrate-responsive element-binding protein–stearoyl-CoA desaturase-1 axis (ChREBP-SCD-1) (43). Further studies to elucidate the signaling pathways and the mechanisms through which ATF3 or ATF3 inducer ST32da regulates the metabolism of brown and beige adipocytes are required. Kim et al. demonstrated that treatment with chalcones, including 2,2,4-trihydroxychalcone and butein, results in vigorous induction of ATF3 and suppression of C3H10T1/2 cell differentiation (44). In summary, these findings indicate that ATF3 plays a coordinating role in inhibiting adipogenesis and lipogenesis, yet to promote beige and brown cell differentiation through white adipocyte browning (Figure 2).

## Role of ATF3 In Immunity

ATF3 induction has been observed in response to a broad range of Toll-like receptors (TLRs), including TLR4, 2/6, 3, 5, 7, and 9 (3). Consequently, ATF3^−/−^ primary macrophages exhibit increased production of IL-6 and IL-12p40 cytokines following TLR activation (3). Gilchrist et al. reported that lipopolysaccharide (LPS) induces IL-6 and IL-12b mRNA levels in bone-marrow-derived macrophages (BMDMs) of ATF3^−/−^ mice. ATF3 interacts with histone deacetylase 1, leading to histone deacetylation and suppression of IL-6 and IL-12b promoter activity in LPS-treated macrophages (45). Thus, ATF3 may negatively regulate the transcription of proinflammatory cytokines containing ATF/CREB binding sites (45). BMDMs show significantly lower survival rates after stimulation with a number of TLR ligands from ATF3-deficient mice compared with WT mice (46). Mechanistically, ATF3 is located downstream of the JNK signaling after TLR stimulation, resulting in repression of pro-apoptotic Bak and Bax transcription (46). ATF3 inhibits LPS-induced chemokine (C-X-C motif) ligand 1 production in mouse airways but promotes neutrophil chemotaxis via T-cell lymphoma invasion and metastasis 2 (TIAM2) expression (47). In addition, basal and LPS-stimulated chemokine (C-C motif) ligand 4 (CCL4) mRNA and protein levels are higher in the BMDMs of ATF3^−/−^ mice compared with those of ATF3^+/+^ mice (4). ATF3 reduces the release of inflammatory molecules, especially high mobility group box 1, which induces lung injury after LPS challenge (48). Furthermore, adeno-associated virus-mediated ATF3 gene transfer could decrease LPS-induced mortality in ATF3^−/−^ mice (48). ATF3^−/−^ mice show inhibited nuclear factor erythroid 2-related factor 2/heme oxygenase-1 signaling, enhanced TLR4-modulated inflammation, and innate cytokine/chemokine gene expression in ischemia/reperfusion-stressed livers (49). ATF3 induction has been observed after interferon (INF) treatment. Type I IFN (IFNα/β) induces ATF3 expression in human and mouse immune cells. ATF3 further regulates a subset of IFN-β-stimulated genes, including CCL12, CCL3, Ch25h, Clec4e (50). Ho et al. found that IFNγ induces ATF3 expression, which conversely binds to the promoter of matrix metalloproteinase 1 (MMP-1), thus reducing its transcription in primary human monocytes and macrophages (51). Interestingly, ATF3 promotes the migration and M1/M2 polarization of RAW 264.7 macrophages by activating tenascin-C via the Wnt/β-catenin signaling pathway (52). During *Streptococcus pneumoniae* infection, pneumolysin induces ATF3 expression via the TLR4/JNK/p38 signaling pathway, which subsequently activates ATF3 and c-Jun hetero-dimerization; the resulting complex then binds to the promoters of cytokines, such as IFN-γ, TNF-α, and IL-1β, leading to increased cytokine production (53). Lee et al. found that ATF3^−/−^ mice show decreased survival and bacterial clearance following *S. pneumoniae* infection. Macrophage ATF3 stimulates IL-17A production in lung γδ T cells to prompt host protection rapidly from early *S. pneumoniae* infection (54). Kim et al. had found that oral treatment of metformin to either mouse with LPS-induced endotoxemia or ob/ob mice decreases the plasma and tissue levels of TNFα and IL-6 and upregulates ATF3 expression in spleen and lungs. In addition, metformin-induced AMPK activation is necessary for ATF3 induction followed by inhibition of LPS-induced MAPK phosphorylation and pro-inflammatory cytokine production in primary peritoneal macrophages. These findings suggest that metformin demonstrates anti-inflammatory action in macrophages partially through pathways involving AMPK activation and ATF3 induction (55).

**Figure 3:**
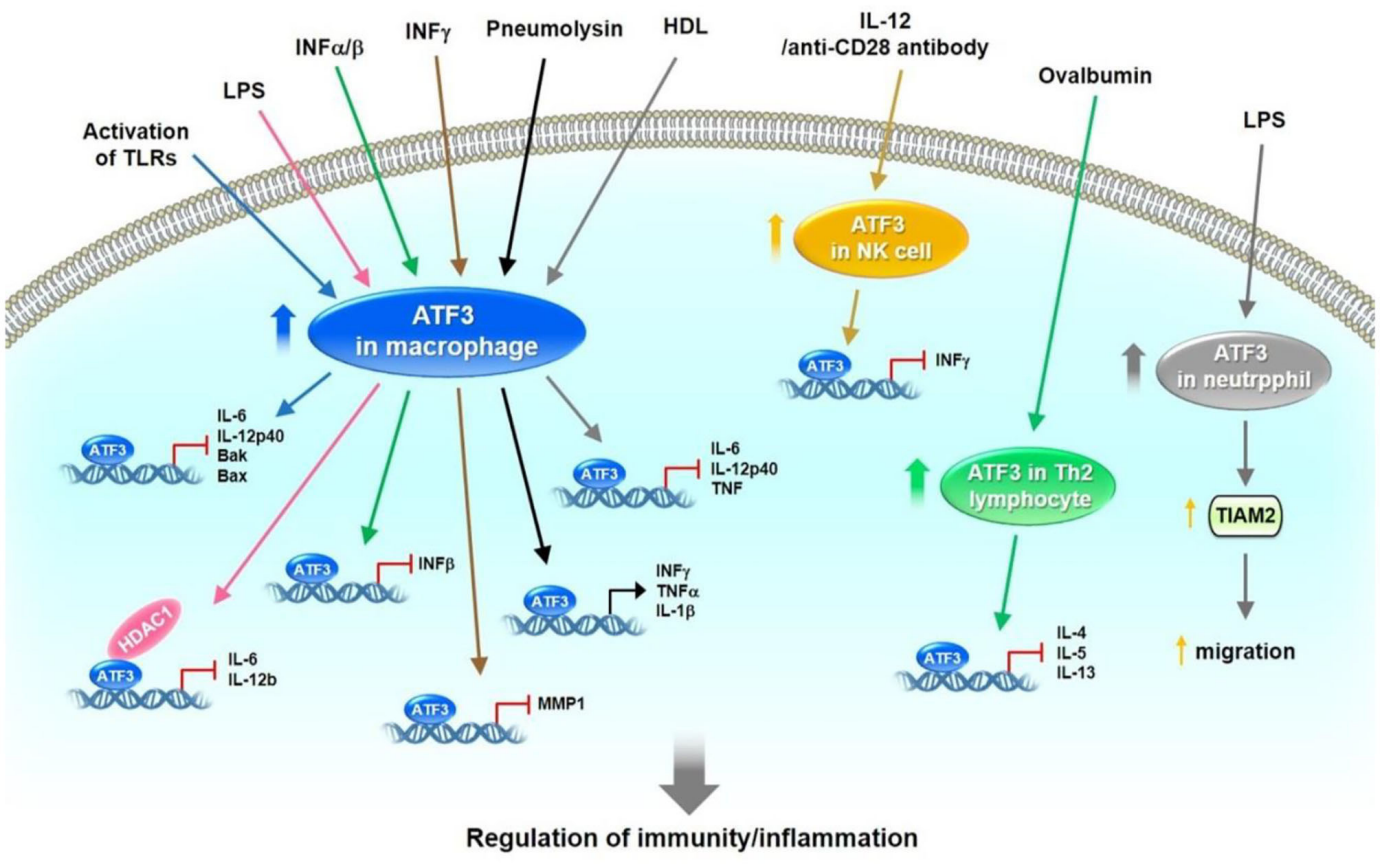
The role of ATF3 in host defense by the regulation of immune responses. In immune cells, such as macrophages, NK, Th2 lymphocyte and neutrophil cells, ATF3 is expressed at low levels. After activation of these immune cells by different signaling pathways initiated by TLRs, LPS, IFNα/β/γ, pneumolysin, IL-12, anti-CD28 antibody and ovalbumin, ATF3 was upregulated. Subsequently, ATF3 downregulates the expression of target genes, including cytokines (IL-1β, IL-4, IL-5, IL-6, IL-12p40, IL-12b, IL-13, TNF, IFNβ/γ) and pro-apoptotic gene (Bak and Bax) by binding to the promoters. In addition, ATF3 enhanced neutrophil chemotaxis via TIAM2 expression Collectively, these observations demonstrate that ATF3 plays an important role in regulating immune responses and maintaining normal host defense.

In contrast to these works, however, some studies indicate that ATF3 acts as a positive regulator for murine cytomegalovirus (MCMV) or bacterial and fungal infections. ATF3^−/−^ mice show increased protection against MCMV infection. ATF3 is upregulated after activation of natural killer (NK) cells by IL-12 and anti-CD28 antibodies (56). Mechanistically, ATF3 negatively regulates IFN-γ expression in NK cells (56). Loss of ATF3 in mice protects against bacterial and fungal infections under reactive oxygen species (ROS) stress (57). ROS superinduces ATF3 expression and suppresses IL-6 production. ATF3^−/−^Il6^−/−^ double-knockout mice are susceptible to infection (57). ATF3 is involved in host immunity against pathogens and some specific inflammatory diseases, such as hepatic steatosis, asthma, and colitis. Liu et al. showed that granulocytic myeloid-derived suppressor cell (G-MDSC) accumulation modulates the effects of glucocorticoids in fatty liver and that ATF3/S100A9 signaling plays a significant role in this process. ATF3 KO mice reveal hepatic steatosis and manifest the expansion of G-MDSCs in the liver and numerous immune organs (58). Enhanced airway hyper-responsiveness and increased chemokine and T-helper 2 (Th2) cytokine responses in lung tissues and lung-derived CD4^+^ lymphocytes have been observed in ATF3 KO mice. Ovalbumin sensitization and challenges result in increased ATF3 transcription in the lungs of WT mice. ATF3 binds to IL-4, IL-5, and IL-13 promoters in Th2 lymphocytes (59). ATF3 expression in CD4^+^ T cells has been negatively correlated with the severity of ulcerative colitis in patients. Deficiency of ATF3 in CD4^+^ T cells aggravates colitis in mice, which could be alleviated by the transfer of follicular helper T (T_FH_) or IgA^+^ B cells. B cell lymphoma 6 (Bcl6) has been identified as a transcriptional target of ATF3 in gut T_FH_ cells (60). A recent study found that ATF3 is involved in the modulation of mucosal immunity through cross-regulation between IL-22-pSTAT3 signaling in gut epithelium cells and IL-6-pSTAT3 signaling in intestinal T-helper 17 (Th17) cells (61). De Nardo et al. found that the protective effects of HDL against TLR-mediated inflammation are dependent on ATF3. ATF3 binds to the promoters of IL-6, IL-12p40, and TNF in the presence of HDL (62). After stress-induced activation of immune cells, ATF3 is upregulated and subsequently downregulates the expression of target genes, including cytokines (e.g., IL-1β, IL-4, IL-5, IL-6, IL-12p40, IL-12b, IL-13, TNF, and IFNβ/γ) and pro-apoptotic genes (e.g., Bak and Bax), by binding to their promoters. In addition, ATF3 enhances neutrophil chemotaxis via TIAM2 expression. These observations show that ATF3 plays a vital role in regulating immune responses and maintaining normal host defense (Figure 3).

## Role of ATF3 in Prostate Cancer

Elevated expression levels of ATF3 have been noted in normal human prostate tissue and the androgen-dependent prostate cancer cell line LNCap, but weak expression levels of ATF3 are observed in androgen-independent cancer cells, including PC3 and DU145 cells. These findings suggest that ATF3 expression could be regulated by androgens (16, 63). Pelzer et al. found that the upregulated expression of ATF3 could be observed in prostate cancer *in vivo* and *in vitro* after androgen stimulation (63). DU-145 transfected with pCMV-ATF3 induces cell proliferation and the G1-to-S-phase transition of the cell cycle (63). Moreover, ATF3 can be SUMOylated, and the main SUMO site for ATF3 is lysine 42 (16). De-SUMOylation decreases ATF3-regulated CCND1 activation in PC3 and DU145 cells. Loss of SUMOylation by transfection with the K42R ATF3 plasmid causes decreased cellular proliferation and colony formation compared with transfection with the WT ATF3 plasmid in PC3 cells (16). ATF3 expression is downregulated by differentiation-related gene-1 (Drg-1), a suppressor of metastasis (64). This observation implies that ATF3 stimulates the metastasis of prostate cancer. Additionally, overexpression of ATF3 fosters the invasiveness and motility of human PC-3MM and ALVA prostate cancer cells. The promotion of pulmonary metastasis in mice was observed after rat ATF3-overexpressing AT2.1 prostate cancer cells were injected subcutaneously into severe combined immunodeficient mice. These findings suggest that ATF3 serves as an oncogene for prostate cancer.

**Figure 4:**
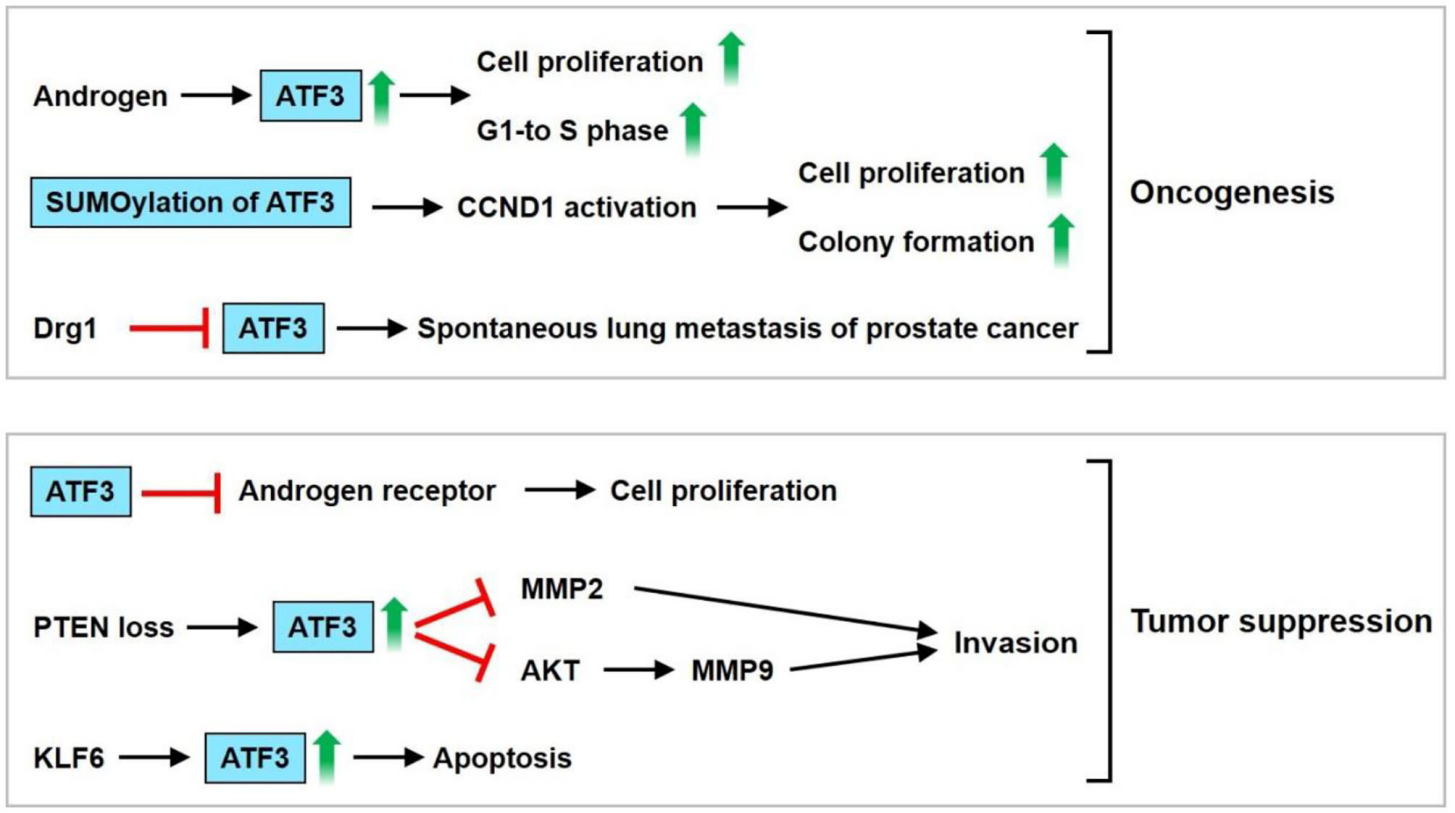
ATF3 functions as an oncogene or tumor suppressor in prostate cancer. ATF3 may be upregulated by androgen, thus induces cell proliferation and G1-to-S-phase transition of the cell cycle. In addition, ATF3 SUMOylation enhanced cyclin D1 (CCND1) activation, resulting in increased cell proliferation and colony formation. ATF3 was downregulated by Drg-1, a suppressor of metastasis. These implied that ATF3 serves an oncogene. ATF3 may act as a tumor suppressor by inhibiting androgen receptor, MMP2, AKT, causing inhibition of cell proliferation and invasion. Furthermore, upregulation of ATF3 by KLF6 leads to cell apoptosis. In sum, these findings demonstrate that dual roles of ATF3 as an oncogene or tumor suppressor is observed in prostate cancer.

However, Wang et al. showed that enhanced prostate epithelial cell proliferation could be observed in ATF3-deficient mice (65). ATF3 suppresses androgen signaling, which is vital for the survival and proliferation of prostate epithelial cells via binding the androgen receptor (AR) (65). ATF3 is induced by phosphatase and tensin homolog deleted on chromosome 10 (PTEN) loss in mouse prostate epithelium and then inhibits AKT activation, leading to reduction of the survival, proliferation, and invasiveness of prostate cancer cells (66). ATF3 is a crucial regulator of Kruppel-like factor 6 (KLF6)-induced apoptosis in prostate cancer cells. Knockdown of KLF6 by siRNA abrogates ATF3 induction and apoptosis under stress conditions, including staurosporine exposure and sodium azide-induced hypoxia (67). These observations suggest that ATF3 serves as a tumor suppressor for prostate cancer. The PI3K and AR signaling pathways are usually activated in prostate cancer. Carver et al. found that a combination of the pharmacologic inhibitor PI3K and AR results in nearly complete prostate cancer regression in a PTEN-deficient prostate cancer mouse model and human prostate cancer xenografts, thereby implying that both signaling pathways equivalently maintain prostate cancer cell survival (68). Udayakumar et al. showed that edelfosine (ET-18-O-CH3), a prototypal synthetic alkyl-lysophospholipid, increases the response of LNCap cells to androgen deprivation (AD) *in vitro* and *in vivo*, thus decreasing tumor cell proliferation and enhancing apoptosis. Furthermore, the combination of ET-18-O-CH3 and AD regulates AR function by suppressing p-AKT and upregulating ATF3 expression (69). In summary, ATF3 may be upregulated by androgens, thus inducing prostate cancer cell proliferation and the G1-to-S-phase transition of the cell cycle. Furthermore, ATF3 SUMOylation enhances CCND1 activation, resulting in increased cell proliferation and colony formation. ATF3 is downregulated by Drg-1. These findings imply that ATF3 serves as an oncogene. However, ATF3 may also act as a tumor suppressor by inhibiting ARs, MMP2, and AKT, thereby causing inhibition of cell proliferation and invasion. The upregulation of ATF3 by KLF6 leads to cell apoptosis. These findings indicate that ATF3 plays dual roles as both an oncogene and tumor suppressor gene in prostate cancer regulation (Figure 4).

## Role of ATF3 in Breast Cancer

ATF3 expression is upregulated in a large number of human breast cancers, possibly owing to the amplification of the ATF3 gene localized within the chromosome 1q amplicon, which is the most commonly amplified region in breast cancer (70). Wang et al. showed that transgenic mice expressing ATF3 (BK5.ATF3 mice) in their basal epithelial cells develop epidermal hyperplasia, oral carcinoma, and mammary carcinoma (71). The canonical Wnt/β-catenin pathway is activated in BK5.ATF3 mammary tumors. Furthermore, downstream Wnt/β-catenin target genes, including CCND1, Jun, Axin2, and Dickkopf 4 (Dkk4), are upregulated in mammary tumors in BK5.ATF3 mice (72). Yin et al. showed that ATF3 expression is upregulated by TGFβ and that ATF3 increases TGFβ gene expression in breast cancer cells, thus forming a positive-feedback loop (73). ATF3 knockdown cells demonstrate decreased cell motility compared with control cells in the presence of TGFβ. Ectopic expression of ATF3 increases the expression of N-cadherin, vimentin, and fibronectin (FN) but decreases E-cadherin to augment epithelial-to-mesenchymal transition (73). Knockdown of ATF-3 expression in MDA-MB231 breast cancer cells decreases cell number, cell migration, and the expression levels of invasive and metastatic genes, such as MMP-13 and Runt-related transcription factor 2 (Runx2) (74). Yin et al. demonstrated that ATF3 exhibits a dual role by stimulating apoptosis in untransformed MCF10A mammary epithelial cells and enhancing cell proliferation, mobility, and invasiveness in malignant MCF10CA1a breast cancer cells (70). In addition, ATF3 upregulates the expression of some genes involved in tumor metastasis, including FN-1, Twist-related protein 1 (TWIST1), plasminogen activator inhibitor-1, urokinase-type plasminogen activator, caveolin-1, and Slug, in MCF10CA1a cells (70).

**Figure 5:**
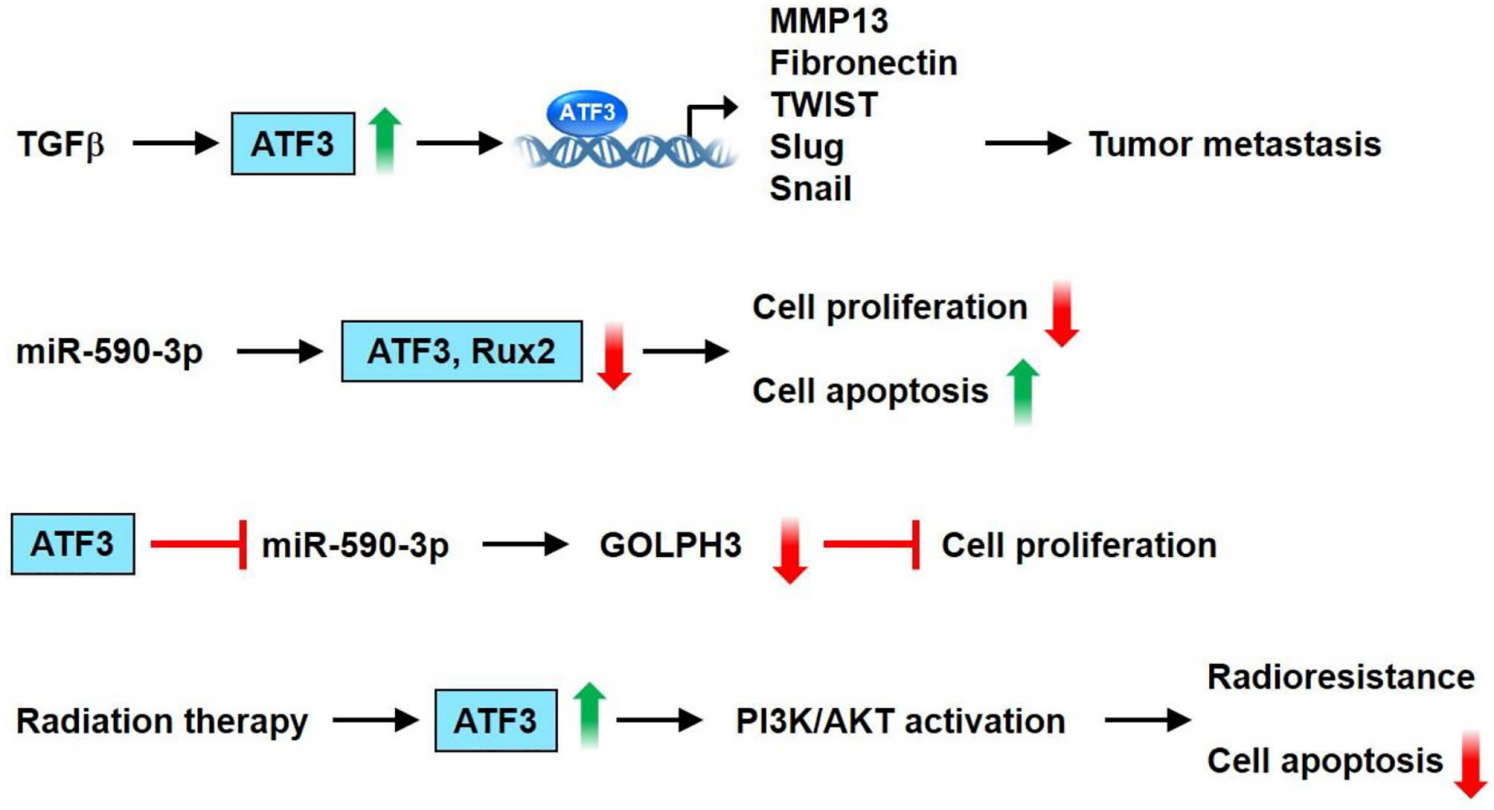
ATF3 plays a vital role in breast cancer. ATF3 expression is increased by TGFβ. Furthermore, ATF3 upregulates the expression of some genes involved in tumor metastasis, including MMP13, fibronectin, TWIST1, Slug and Snail in the MCF10CA1a cells. Overexpression of miR-590-3p decreased cell proliferation and increased apoptosis via downregulation of the expression of ATF3 and Runx2 in MDA-MB231 cells. In addition, ATF-3 inhibits miR-590-3p expression to regulate miR-590/GOLPH3 pathway, and thus modulates proliferation of breast cancer cells. ATF3 was shown to be involved in radioresistance of breast cancer via PI3K/AKT signaling pathway. These observations show that ATF3 acts as an important regulator in breast cancer.

The ATF3 gene is amplified and increased in human breast cancer (70). ATF-3 is highly upregulated and its expression level is maintained by TGF-ß1 in highly invasive/metastatic human breast cancer (MDA-MB231) (75). Transient transfection of the ATF3 shRNA construct into MDA-MB231 cells results in a downregulated expression of the cell cycle gene, cyclin A1, and an invasive and metastatic gene, MMP-13 (75). Besides cancer cells, the tumor microenvironment consists of diverse cell types, including endothelial cells, immune cells, and fibroblasts. ATF3 is upregulated in the stromal compartment of several types of cancer, including colon, lung, and breast cancers (76). Cancer-associated fibroblasts overexpressing ATF3 demonstrate rapid proliferation, as indicated by their enhanced colony-forming ability and fast growth in adjacent tumor cells, when co-injected into nude mice (76). ATF3 expression can be modulated by post-transcriptional regulators such as microRNAs. Rohini et al. showed that overexpression of miR-590-3p downregulates the expression of ATF3 and Runx2 in MDA-MB231 cells. Moreover, overexpression of miR-590-3p decreases proliferation and increases the apoptosis of breast cancer cells (77). Using publicly available RNA array datasets (78, 79), Wolford et al. found that high ATF3 expression in breast cancer epithelium cells is correlated with decreased survival and lower metastasis-free survival (80). Further immunohistochemical analysis of patient tumor samples showed that ATF3 expression in stromal mononuclear cells is correlated with poor clinical outcomes. ATF3 KO mice show less efficient breast cancer metastasis than WT mice. Furthermore, mice with myeloid cell-selective KO of ATF3 revealed reduced lung metastases (80). Cao et al. demonstrated through immunohistochemical detection that ATF3 protein is upregulated in breast cancer tissue compared with negative control samples and adjacent normal breast tissues (81). A significant correlation between ATF3 expression level and tumor-node-metastasis stage/lymph node metastasis/invasion/number of metastatic lymph nodes was also noted (81). Chang et al. showed that the pro-cancer effects of the cancer chemotherapeutic agent paclitaxel, such as enhanced tumor microenvironment of metastasis, inflammatory monocytes, and metastasis, depend on ATF3 in the non-cancer host cells (82). In a spontaneous metastasis mouse model produced by fat-pad injection of cancer cells, paclitaxel remarkably aggravated breast cancer metastasis in WT mice but only slightly affected ATF3 KO mice. The genotype difference between the mice demonstrated that the processes regulated by ATF3 in the host cells are key regulators through which paclitaxel aggravates metastasis (82). ATF3 expression is upregulated in breast cancer after radiation therapy, and the transcription factor has been involved in the radio-resistance of breast cancer through the PI3K/AKT signaling pathway (83). Hasim et al. demonstrated that ATF3 upregulation plays a remarkable role in regulating the cytotoxicity of doxorubicin in breast cancer treatment. This study further found that the combination of ATF3-inducing agents (e.g., trifluridine or vorinostat) with doxorubicin may be utilized as a novel therapeutic application for this type of cancer (84). Song et al. showed the signaling mechanisms through which ATF3 is involved in the miR590-mediated cell proliferation of breast cancer (85). The authors found that miR-590 targets Golgi phosphoprotein 3 (GOLPH3) to suppress breast cancer cell proliferation. Furthermore, ATF-3 inhibits miR-590-3p expression to modulate the miR-590/GOLPH3 pathway and, thus, regulate the proliferation of breast cancer cells (85). In summary, ATF3 can upregulate genes involved in breast cancer cell metastasis, including MMP13, FN, TWIST1, Slug, and Snail, in MCF10CA1a cells. Overexpression of miR-590-3p decreases cancer cell proliferation and increases apoptosis via downregulation of the expression of ATF3 and Runx2 in MDA-MB231 cells. Furthermore, ATF-3 inhibits miR-590-3p expression to regulate the miR-590/GOLPH3 pathway and, thus, modulates breast cancer cell proliferation. ATF3 was shown to be involved in radio-resistance of breast cancer via the PI3K/AKT signaling pathway. These observations suggest that ATF3 acts as an important stimulatory regulator of breast cancer cell proliferation and tumor metastasis (Figure 5).

## Role of ATF3 in Colon Cancer

The effects of ATF3 expression are especially complicated in colon cancer. ATF3 expression was reportedly downregulated in the tumors as compared with their normal surrounding tissue (86, 87). Bottone et al. showed that ATF3 overexpression could inhibit the migration/invasion of the HCT116 cells and decrease the size of the mouse tumor xenografts (88). In addition, altered expression of invasion-related genes, such as mammary serine protease inhibitor (maspin), plasminogen activator inhibitor-1 (PAI-1), β-catenin, and metastasis-associated 1 (MTA-1) could be observed in ATF3 overexpressing HCT116 cells (88). ATF3 promotes death receptor 5 (DR5) induction and apoptotic cell death upon zerumbone (a bioactive sesquiterpene) or celecoxib (a selective inhibitor of cyclooxygenase 2) treatment in human p53-deficient colorectal cancer cells (89). ATF3 enhances sensitization of human colorectal cancer cells to TNF-related apoptosis-inducing ligand (TRAIL)-mediated apoptosis through endoplasmic reticulum (ER) stress (89). Taketani et al. reported that the DR5 gene is a direct target of ATF3 upon DNA damage, and ATF3 plays a key role in apoptosis induced by camptothecin (CPT) /TRAIL co-treatment of human colorectal cancer cells (90). Some studies showed that heat-shock protein 90 (Hsp90) inhibitor (17-DMAG) induces ATF3 expression in human gastric (TMK-1), colon (HT29, HCT116, SW620), and pancreatic (L3.6pl) cancer cell lines (87, 91). Down-regulation of ATF3 by ATF3-shRNA causes an increased tumor growth rate of HCT116 colon cancer cells as compared with control cells (87). Several studies show that non-steroidal anti-inflammatory drugs (NSAIDs) such as sulindac have anti-tumorigenic effects in colorectal cancer (92–94). ATF3 is upregulated by treatment with cyclooxygenase (Cox) inhibitor (sulindac sulfide) or Cox-1 specific inhibitor SC-560 or Cox-2 specific inhibitor SC-58125, suggesting that ATF3 may play a critical role in sulindac sulfide/SC-560/SC-58125-induced apoptosis and anti-tumorigenic effect in human colorectal cancer cells HCT-116 and SW-480 (86, 95). In addition to Cox inhibitors, numerous dietary compounds such as resveratrol and genistein could inhibit cell proliferation of HCT-116 cells and are reported to upregulate ATF3 expression (96). Naringenin (NAR) as one of the flavonoids found in grapefruit has been reported to have anti-cancer activity. NAR upregulates ATF3 expression in HCT116, SW480, LoVo, and HT-29 colon cancer cells. Furthermore, ATF3 overexpression enhances NAR-modulated cleavage of PARP and ATF3 knockdown decreases PARP cleavage by NAR. These findings suggest that ATF3 may be one of the molecular targets for NAR-modulated apoptosis in colon cancer cells (97). *Trans*-10, *cis*-12-conjugated linoleic acid (CLA) could increase ATF3 expression through transcriptional upregulation of the ATF3 gene. In addition, ATF3 modulates *trans-10, cis-12-*CLA-stimulated non-steroidal anti-inflammatory drug-activated gene-1 (NAG-1) expression, and apoptosis in colon cancer cells (98). These observations suggest that ATF3 acts as a tumor suppressor for colon cancer.

**Figure 6:**
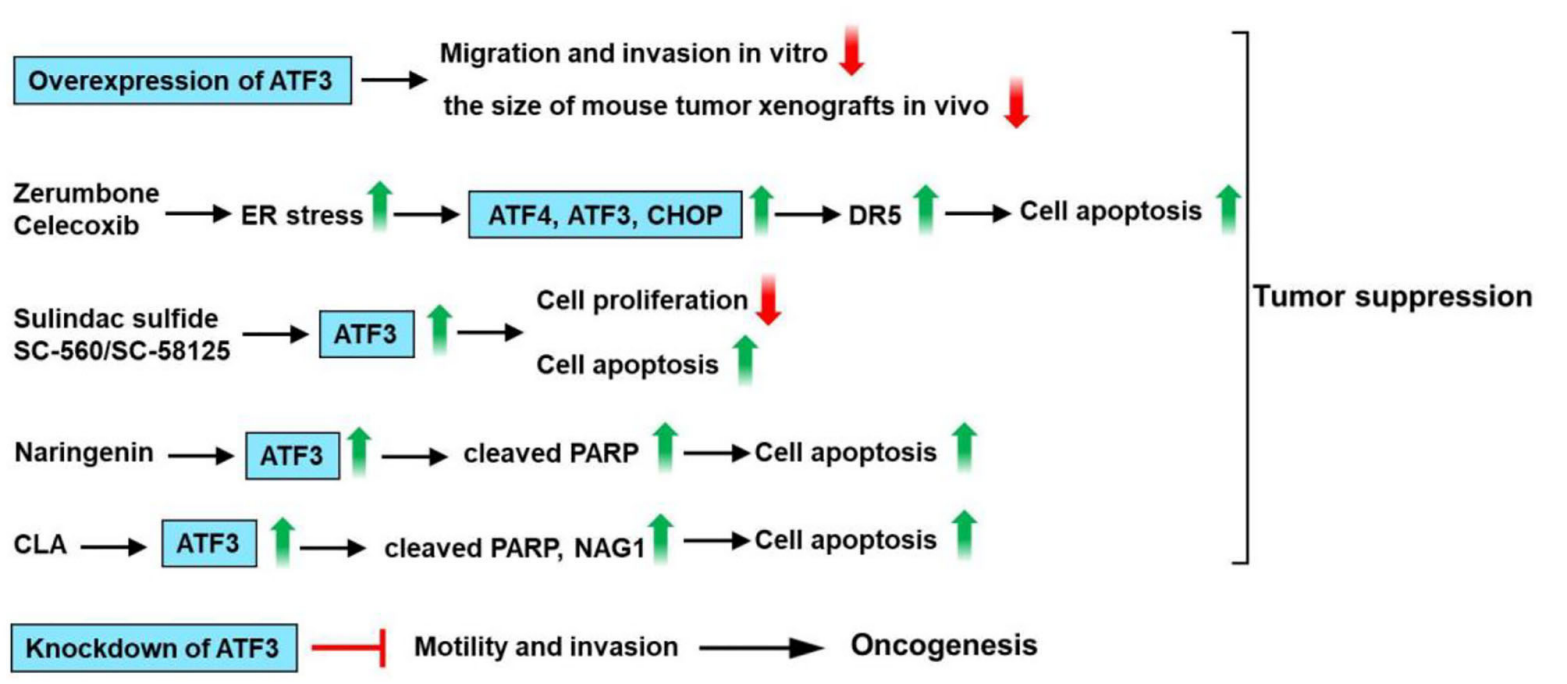
ATF3 plays a critical role in colon cancer. ATF3 overexpression could inhibit the migration and invasion of the HCT116 cells and decrease the size of mouse tumor xenografts. ATF3 promotes death receptor 5 (DR5) induction and apoptotic cell death upon zerumbone (a bioactive sesquiterpene) or celecoxib (a selective inhibitor of cyclooxygenase 2) treatment in human p53-deficient colorectal cancer cells. ATF3 is upregulated by treatment with cyclooxygenase (Cox) inhibitor (sulindac sulfide) or Cox-1 specific inhibitor SC-560 or Cox-2 specific inhibitor SC-58125, suggesting that ATF3 may be involved in sulindac sulfide/SC-560/SC-58125-induced apoptosis and anti-tumorigenic effect in human colon cancer cells. Naringenin (NAR) upregulates ATF3 expression in colon cancer cells. Furthermore, ATF3 overexpression enhances NAR-modulated cleavage of PARP. Trans-10, cis-12-conjugated linoleic acid (CLA) could increase ATF3 expression. In addition, ATF3 modulates trans-10, cis-12-CLA-stimulated non-steroidal anti-inflammatory drug-activated gene-1 (NAG-1) expression and apoptosis in colon cancer cells. These findings suggest that ATF3 acts as a tumor suppressor for colon cancer. However, siRNA-mediated knockdown of ATF3 attenuates motility and invasion of the colon cancer cell. These observations suggest that ATF3 plays a dual role in colon cancer.

However, several studies indicated that ATF3 expression is upregulated in human colon cancer specimens and promotes tumor growth and migration in HT29 colon cancer cells (99–101). Ishiguro et al. had shown that the ATF3 antisense oligonucleotide inhibited cell adhesion and invasion as compared with the control cells (100). In addition, siRNA-mediated knockdown of ATF3 attenuates motility and invasion of the colon cancer cell lines HT29 and CaCO_2_ (101). These findings suggest that ATF3 serves as an oncogene for colon cancer. In summary, ATF3 promotes DR5 induction and apoptotic cell death upon zerumbone or celecoxib treatment in human p53-deficient colorectal cancer cells. ATF3 is upregulated by treatment with sulindac sulfide or SC-560 or SC-58125 and involved in sulindac sulfide/SC-560/SC-58125-induced apoptosis and anti-tumorigenic effect. Furthermore, ATF3 overexpression enhances the NAR-modulated cleavage of PARP. ATF3 modulates trans-10, cis-12-CLA-stimulated NAG-1 expression, and apoptosis. However, siRNA-mediated knockdown of ATF3 attenuates motility and invasion of the colon cancer cell. These observations suggest that ATF3 plays a dual role in colon cancer (Figure 6).

## Role of ATF3 in Lung Cancer

**Figure 7:**
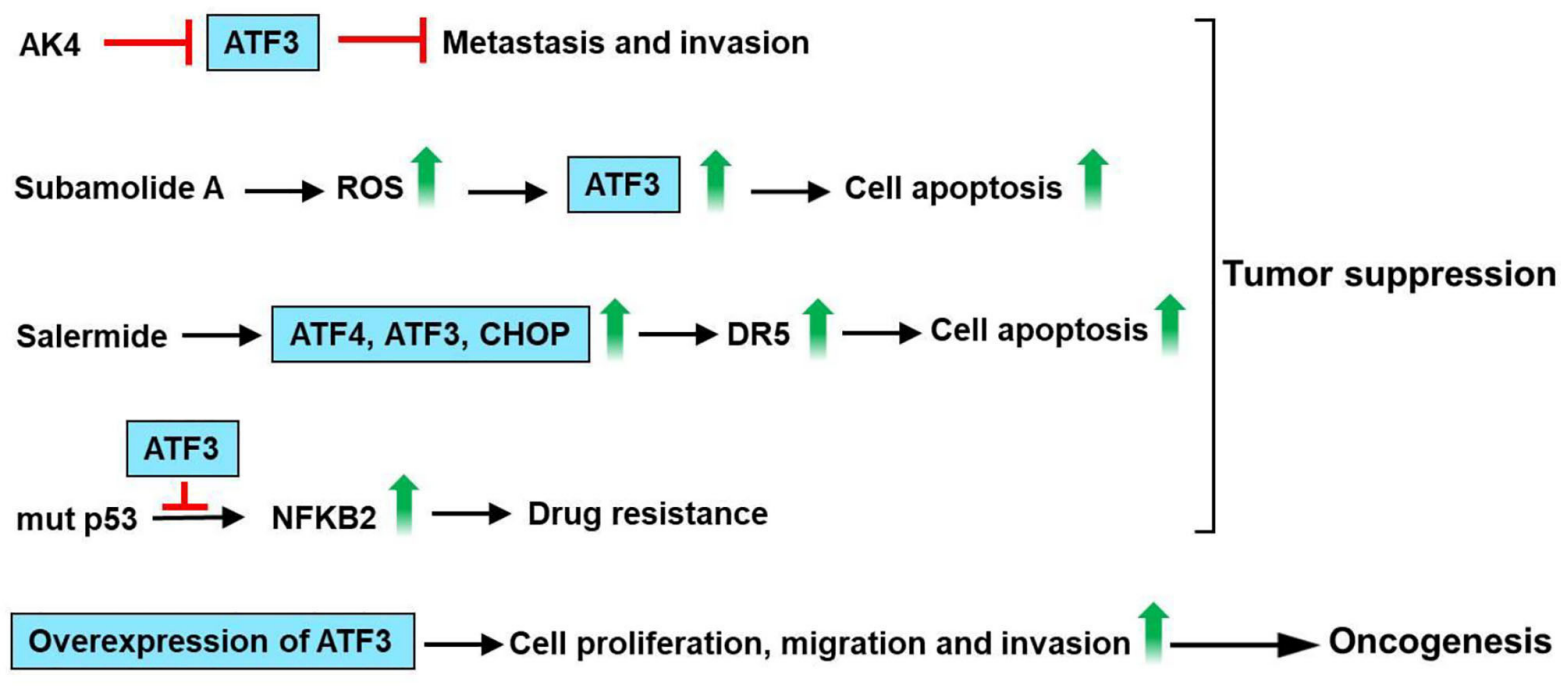
ATF3 plays an important role in lung cancer. ATF3 was identified as a vital regulatory target of adenylate kinase-4 (AK4). AK4 stimulates metastasis of lung cancer by downregulation of ATF3 expression. Subamolide A induced mitotic catastrophe and apoptosis, mainly via ROS upregulation that induces ataxia-telangiectasia mutation (ATM) and ATF3 activation, subsequently resulting in p53-mediated cell death. In addition, salermide upregulates ATF3 expression and triggers ER stress in human non-small cell lung cancer (NSCLC) cells, which regulates the induction of the ATF4-ATF3-C/EBP homologous protein (CHOP) axis that results in DR5-dependent apoptosis. ATF3 decreases nuclear factor kappa B subunit 2 (NFKB2) expression and reverse drug resistance caused by mutant p53 protein in H1299 cells. These findings suggest that ATF3 acts as a tumor suppressor for lung cancer. However, knockdown of ATF3 expression by RNA interference significantly inhibited the cell proliferation, migration, and invasion in A549 and H1299 cells. These observations show that ATF3 acts as an important regulator in lung cancer.

ATF3 is upregulated by cisplatin and carboplatin in lung cancer cells (A549), human ovarian cancer cells (SKOV-3, A2780-cp), breast cancer cells (MCF-7) and prostate cancer cells (PC3) (102). ATF3 expression mediates, in part, the cisplatin-modulated apoptosis in lung cancer (102). Jan et al. had found that ATF3 was identified as a critical regulatory target of adenylate kinase-4 (AK4). Furthermore, AK4 is a marker of poor clinical outcomes that stimulates the metastasis of lung cancer by downregulation of ATF3 expression (103). Human lung cancer cells A549 and NCIH460 are vastly sensitive to subamolide A (isolated from *Cinnamomum subavenium*)-induced mitotic catastrophe and apoptosis, mainly via ROS upregulation that induces ataxia-telangiectasia mutation (ATM) and ATF3 activation, subsequently resulting in p53-mediated cell death (104). Liu et al. have found that salermide (a novel histone deacetylase) upregulates ATF3 expression. Furthermore, salermide triggers ER stress in human non-small cell lung cancer (NSCLC) cells, which modulates the induction of the ATF4-ATF3-C/EBP homologous protein (CHOP) axis that results in DR5-dependent apoptosis (105). It is reported that ATF3 interacts with mutant p53 protein in H1299 NSCLC cells. In addition, ATF3 decreases nuclear factor kappa B subunit 2 (NFKB2) expression and reverse drug resistance caused by mutant p53 protein in H1299 cells (106). These observations suggest that ATF3 acts as a tumor suppressor for lung cancer. However, increased ATF3 expression was observed in lung cancer tissues/cells as compared with normal tissues/cells. The knockdown of ATF3 expression by RNA interference significantly inhibited cell proliferation, cell cycle progression, migration, and invasion in A549 and H1299 cells (107). These observations suggest that ATF3 acts as an oncogene for lung cancer. In summary, AK4 stimulates metastasis of lung cancer by downregulation of ATF3 expression. Subamolide A-induced mitotic catastrophe and apoptosis, mainly via ROS upregulation that induces ATM and ATF3 activation, subsequently resulting in p53-mediated cell death. Salermide upregulates ATF3 expression and triggers ER stress, which regulates the induction of the ATF4-ATF3-CHOP axis that results in DR5-dependent apoptosis. ATF3 decreases NFKB2 expression and reverse drug resistance caused by mutant p53 protein in H1299 cells. However, knockdown of ATF3 expression significantly inhibited cell proliferation, migration, and invasion in A549 and H1299 cells. These observations show that ATF3 acts as an important regulator in lung cancer (Figure 7).

## Role of ATF3 in Liver Cancer

**Figure 8:**
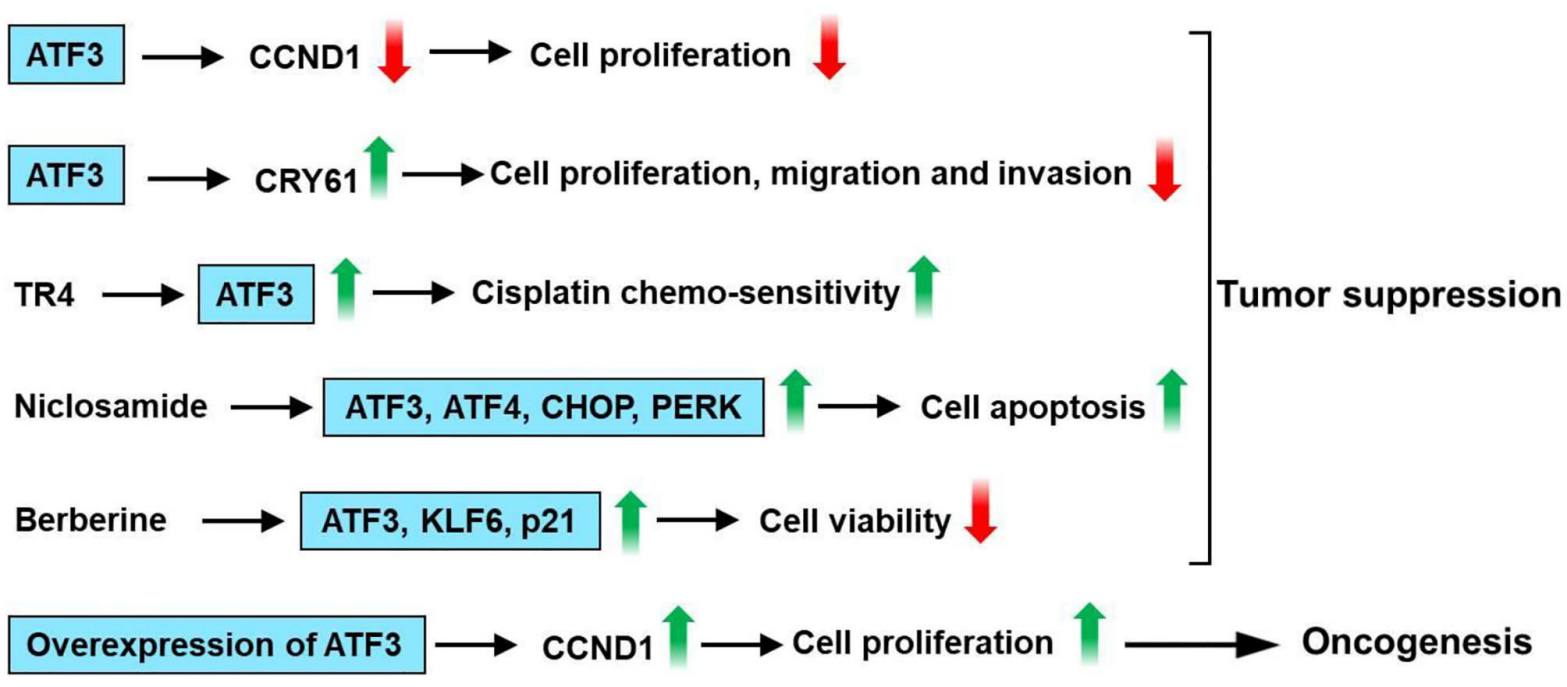
ATF3 plays a vital role in liver cancer. Overexpression of ATF3 downregulates cyclin D1 expression in HepG2 cells. Cysteine-rich angiogenic inducer 61 (CYR61) is involved in the inhibitory effect of ATF3 on HCC cells. Knockdown of CYR61 significantly reversed ATF3-induced suppression of cell proliferation, migratory and invasive abilities in HCC cells. ATF3 is involved in testicular receptor 4 (TR4) upregulation of the cisplatin chemo-sensitivity in HCC cells. Niclosamide upregulates ATF3, ATF4, CHOP, and PRKR-like endoplasmic reticulum kinase (PERK) expression in HCC cells. ATF3 knockdown HCC cells had higher cell viability than control cells after niclosamide treatment. Berberine (BBR) suppresses proliferation and migration as well as induces cell cycle arrest and apoptosis in HCC cells. Furthermore, BBR upregulates protein expression of tumor suppressor genes, including KLF6, ATF3, and p21. These observations suggest that ATF3 acts as a tumor suppressor for liver cancer. However, overexpression of ATF3 increases cyclin D1 mRNA expression in the Hepa 1–6 mouse hepatoma cells. These observations show that ATF3 plays a dual role in liver cancer.

ATF3 level is low in human hepatocellular carcinoma (HCC) tissues (108, 109), and there was a decreased ATF3 protein level in patients with capsule invasion (108). Li et al. had shown that overexpression of ATF3 inhibits growth, decreases cell cycle progression, and increases the apoptotic activity of HepG2 cells (110). Overexpression of ATF3 downregulates cyclin D1 expression in HepG2 cells (109). In addition, there was a negative correlation between ATF3 and cyclinD1 mRNA expression in HCC samples (109). ATF3 inhibited cell proliferation in HCC cells, including SK-Hep1, Li-7, MHCC-LM3, and MHCC-97H cells and HCC cell tumorigenesis *in vivo* (111). Mechanistically, cysteine-rich angiogenic inducer 61 (CYR61) is involved in the inhibitory effect of ATF3 on HCC cells. The knockdown of CYR61 significantly reversed ATF3-induced suppression of cell proliferation, migratory, and invasive abilities in HCC cells (111). It is reported that ATF3 is involved in testicular receptor 4 (TR4) upregulation of the cisplatin chemo-sensitivity in HCC cells (112). TR4 directly binds to the promoter of ATF3 through the ChIP assay (112). Weng et al. had found that niclosamide, an antihelminthic drug, induced cell apoptosis in HCC cells (113). Furthermore, niclosamide upregulates ATF3, ATF4, CHOP, and PRKR-like endoplasmic reticulum kinase (PERK) expression in HCC cells. ATF3 knockdown HCC cells had higher cell viability than control cells after niclosamide treatment (113). Berberine (BBR), an isoquinoline alkaloid, which can be isolated from several naturally occurring plants such as *Phellodendron chinense schneid, Coptidis rhizoma*, and *Phellodendron amurense* (114). BBR suppresses proliferation and migration as well as induces cell cycle arrest and apoptosis in HCC cells (115, 116). Furthermore, BBR upregulates protein expression of tumor suppressor genes, including KLF6, ATF3, and p21 (116). These observations suggest that ATF3 acts as a tumor suppressor for liver cancer. However, overexpression of ATF3 increases DNA synthesis and cyclin D1 mRNA expression in the Hepa 1–6 mouse hepatoma cells (117). Additionally, Lin et al. had found that cAMP-response element-binding protein 2 (CREB-2) and ATF3 have an oncogenic role as transcriptional activators of signal transducer and activator of transcription 3 (STAT3) in liver cancer formation when spectrin beta, non-erythrocytic 1 (SPTBN1), and/or SMAD3 fail to function (118). In summary, overexpression of ATF3 downregulates cyclin D1 expression in HepG2 cells. Knockdown of CYR61 reversed ATF3-induced suppression of cell proliferation, migratory, and invasive abilities in HCC cells. ATF3 is involved in TR4 upregulation of the cisplatin chemo-sensitivity in HCC cells. Niclosamide upregulates the expression of ATF3, ATF4, CHOP, and PERK in HCC cells. ATF3 knockdown HCC cells had higher cell viability than control cells after niclosamide treatment. BBR suppresses proliferation and migration as well as induces cell cycle arrest and apoptosis. The protein expression of tumor suppressor genes, including KLF6, ATF3, and p21 was upregulated by BBR. However, overexpression of ATF3 increases cyclin D1 mRNA expression in the Hepa 1–6 mouse hepatoma cells. These observations show that ATF3 plays a dual role in liver cancer (Figure 8).

## Conclusions

ATF3 plays an important role in modulating immune homeostasis, glucose and adipose tissue regulation, and cell proliferation and metastasis in breast, prostate, colon, lung, and liver cancers. As a master regulator of metabolic homeostasis, ATF3 decreases the expression of GLUT4 and adiponectin in adipose tissue but suppresses gluconeogenesis in the liver. Although the regulation of pancreatic glucose homeostasis by ATF3 remains debatable, the transcription factor has been shown to modulate glucose homeostasis by regulating food intake and energy metabolism in the hypothalamus. In addition, ATF3 regulates adipocyte metabolism by modulating adipogenic/lipogenic/browning/beige gene expression. ATF3 plays a repressive role in stress-induced immune responses. Recent studies indicate that ATF3 may play a dual role as an oncogene and tumor suppressor depending on the tissue condition. The transcription factor is important for two host defenses, including immunity against pathogens and cancer progression. Obtaining a better understanding of how ATF3 modulates metabolic, immuno-responsive, and oncogenic signaling pathways could pave the way for the transcription factor to become an intriguing target for the treatment of metabolic dyshomeostasis, immune disorders, and various cancers. Using tissue-specific gain- or loss-of-function methods is crucial to elucidate the role of ATF3 in the regulation of immune homeostasis, cancer, and glucose and adipose tissue metabolism.

## Author Contributions

All authors listed have made a substantial, direct and intellectual contribution to the work, and approved it for publication.

## Conflict of Interest

The authors declare that the research was conducted in the absence of any commercial or financial relationships that could be construed as a potential conflict of interest.
